# A brain-wide functional map of the serotonergic responses to acute stress and fluoxetine

**DOI:** 10.1038/s41467-018-08256-w

**Published:** 2019-01-21

**Authors:** Joanes Grandjean, Alberto Corcoba, Martin C. Kahn, A. Louise Upton, Evan S. Deneris, Erich Seifritz, Fritjof Helmchen, Edward O. Mann, Markus Rudin, Bechara J. Saab

**Affiliations:** 1 Singapore Bioimaging Consortium, Agency for Science Technology and Research, 11 Biopolis Way, Singapore, 138667 Singapore; 2 Neuroscience Center Zurich, Winterthurerstr. 190, CH-8057 Zurich, Switzerland; 3 Institute for Biomedical Engineering, University of Zurich and ETH Zurich, CH-8093 Zurich, Switzerland; 4 Center for Psychiatric Neuroscience, University of Lausanne, CH-1008 Prilly-Lausanne, Switzerland; 5 Preclinical Laboratory for Translational Research into Affective Disorders, Department of Psychiatry, Psychotherapy and Psychosomatics, University of Zurich Hospital for Psychiatry, August-Forel-Str. 7, CH-8008 Zurich, Switzerland; 6 Department of Physiology, Anatomy and Genetics, University of Oxford, Oxford, OX1 3PT UK; 7 Oxford Ion Channel Initiative, University of Oxford, Oxford, OX1 3PT UK; 8 Department of Neurosciences, Case Western Reserve University School of Medicine, 10900 Euclid Ave., Cleveland, OH 44106-4975 USA; 9 Brain Research Institute, University of Zurich, Winterthurerstr. 190, CH-8057 Zurich, Switzerland; 10 Institute of Pharmacology and Toxicology, University of Zurich, CH-8093 Zurich, Switzerland; 11 Present Address: Mobio Interactive, Thurwiesenstrasse 4, CH-8037 Zurich, Switzerland

## Abstract

Central serotonin (5-HT) orchestrates myriad cognitive processes and lies at the core of many stress-related psychiatric illnesses. However, the basic relationship between its brain-wide axonal projections and functional dynamics is not known. Here we combine optogenetics and fMRI to produce a brain-wide 5-HT evoked functional map. We find that DRN photostimulation leads to an increase in the hemodynamic response in the DRN itself, while projection areas predominately exhibit a reduction of cerebral blood volume mirrored by suppression of cortical delta oscillations. We find that the regional distribution of post-synaptically expressed 5-HT receptors better correlates with DRN 5-HT functional connectivity than anatomical projections. Our work suggests that neuroarchitecture is not the primary determinant of function for the DRN 5-HT. With respect to two 5-HT elevating stimuli, we find that acute stress leads to circuit-wide blunting of the DRN output, while the SSRI fluoxetine noticeably enhances DRN functional connectivity. These data provide fundamental insight into the brain-wide functional dynamics of the 5-HT projection system.

## Abstract (web summary)

Serotonin is released throughout the brain from diverse projections of neurons in the dorsal raphe nucleus. Here, the authors use optogenetics and fMRI mediated mapping of the mouse brain-wide serotonin network in response to acute stress and treatment with SSRI.

## Introduction

The central nervous system (CNS)’s expansive serotonergic (5-HT) circuit is amongst the most versatile and important neurotransmitter systems for emotional and cognitive processing. Primarily originating within the phylogenetically ancient dorsal raphe nucleus (DRN), a small brainstem nuclei that projects promiscuously throughout the brain, the CNS 5-HT circuit influences mood, memory, circadian rhythm, feeding, feeling of reward and stress coping, and is strongly implicated in the aetiology and treatment of many prevalent neurological disorders, especially those related to stress^1–4^. However, much remains unknown about its basic character, particularly with respect to its functional connectivity. For example, are the neural correlates of hemodynamic responses from 5-HT signalling the same as those for glutamatergic signalling? Does the anatomical architecture of the 5-HT circuit correlate with its functional connectivity? How do separate stimuli that lead to elevated synaptic 5-HT, e.g. selective serotonin reuptake inhibitor (SSRI) and acute stress, affect the elicited circuits?

To address these outstanding questions, we used optogenetic combined with functional magnetic resonance imaging (ofMRI)^5^ to establish a whole-brain visualisation of the central 5-HT functional circuit in the live mouse. We find the functional map to be indicative of bidirectional circuit regulation, and its functional connectivity to better match regional expression of certain 5-HT receptor subtypes than 5-HT neuron projection density. In addition, we find that delta oscillations, more so than gamma oscillations or multi-unit activity (MUA), best mirror hemodynamic changes associated with optogenetically evoked 5-HT release across the cortex. When examining the circuit following either acute stress or administration of fluoxetine, we observe opposite effects on DRN 5-HT functional connectivity, providing an elegant explanation at the circuit level for the behavioural divergence of these stimuli. Our observations underscore the power of ofMRI for characterizing large brain networks originating from subcortical nuclei, as well for analysing effects of acute stimuli on neuromodulatory systems.

## Results

### A whole-brain functional map of the DRN 5-HT circuit

**Fig. 1:**
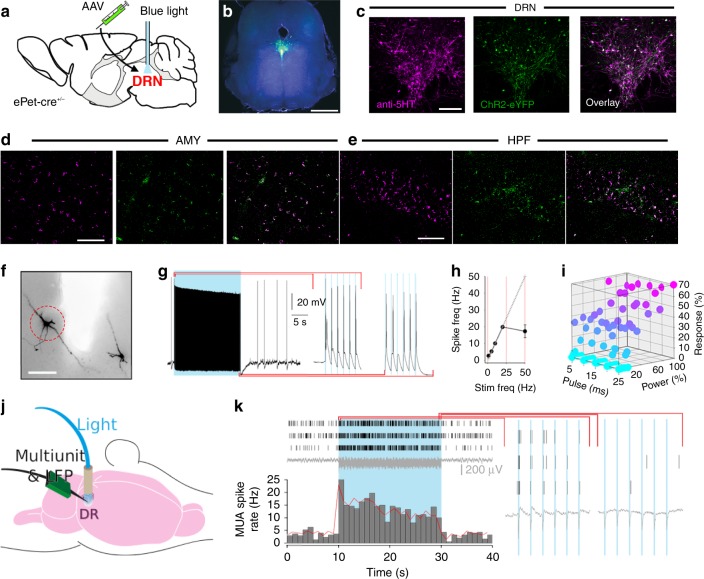
Optogenetic targeting of DRN 5-HT neurons. **a** Infusion of AAV harbouring cre-dependent ChR2-eYFP into the DRN of *ePet-cre*^*+/−*^ mice, followed by implantation of a MRI-friendly optic fibre used to deliver blue light. **b** Whole midbrain slice with Ch2R in green. DAPI (blue) identify all cell nuclei. Scale bar indicates 2.5 mm. **c**–**e** Co-immunofluorescence with anti-5-HT (purple) and ChR2-eYFP (green) in the (**c**) DRN, (**d**) amygdala (AMY) and (**e**) hippocampus (HPF). Scale bars indicate 500, 100 and 50 µm, respectively. **f** Biocytin-filled neurons of the DRN used for in vitro whole-cell patch electrophysiology. Red dashed circle indicates recorded neuron. Scale bar indicates 200 µm. **g** Response of neuron circled in (**f**) to all but one pulse of a blue light train. **h** Frequency response curve from midbrain slices. Linearity stable to 20 Hz. **i** Strength of response expressed in maximum number of spikes per pulse during 20 Hz photostimulation, as a function of pulse width and laser power. **j** Schematic of the experimental set-up for paired MUA and LFP recordings in the DRN during photostimulation. **k** Raster plots of MUA activity in three neighbouring channels (top), LFP (middle) and mean spike histogram (bottom), revealing activation of the DRN 5-HT network during a blue light train (20 Hz, 5 ms pulse width). Expanded to the right are the MUA and LFP for the first and last 6 pulses in the train

To identify and control the activity of midbrain neurons expressing *Pet-1*, a gene critical for 5-HT neuron development and function, we injected a Cre-inducible viral construct encoding Channelrhodopsin-2 fused to enhanced yellow fluorescent protein (ChR2-eYFP) into the DRN of *ePet-Cre*^*+/−*^ mice (Fig. 1a). In these *ePet*^*DRN*^*::ChR2-eYFP* mice, 96.1 ± 0.8% (mean ± 1 standard deviation) of ChR2-expressing DRN neurons co-stained for 5-HT, demonstrating highly specific targeting of serotonergic neurons, with 75.4 ± 5.4% of 5-HT-immunopositive neurons coexpressing ChR2-eYFP (*n* = 3 mice; Fig. 1b, c, Supplementary Figure 1). While no ChR2-eYFP-positive cell bodies were found outside the DRN (Fig. 1b, c), axons co-expressing ChR2-eYFP and 5-HT were found terminating in the neocortex (Supplementary Figure 2), thalamus (Supplementary Figure 3), amygdala (Fig. 1d; Supplementary Figure 4), dorsal hippocampus (Fig. 1e; Supplementary Figure 5) and striatum (Supplementary Figure 6). Consistent with earlier reports, these data highlight the structural substrate by which midbrain-derived serotonin neurons could modulate nearby and distant brain regions^2^. To ascertain the efficacy of the opsin, we performed electrical recordings during blue light illumination in vitro and found neurons stimulated with a 5 ms pulse remained faithful to a 20 s, 20 Hz stimulus train, even in the presence of anaesthetic (Fig. 1f–i, Supplementary Figure 7). This paradigm was found to maximise spiking recorded within the DRN while minimizing duty cycle. Block lengths was optimized for ofMRI detection to correspond to 10 volumes in our acquisitions. Importantly, the paradigm was selected to induce contrasting level of activity relative to baseline and may not reflect DRN physiological activity.

To determine whether the activity of serotonergic neurons affect target structure cell activity, we combined in vivo multiunit recordings with photostimulation in 5 *ePet*^*DRN*^*::ChR2-eYFP* mice and 2 *WT*^*DRN*^*::ChR2-eYFP* control mice anaesthetised using an anaesthesia regime optimised for small animal functional magnetic resonance imaging (fMRI) (0.5% isoflurane + 0.2 mg/kg/h s.c. medetomidine)^6^. In *ePet*^*DRN*^*::ChR2-eYFP* mice, the firing rate of DRN neurons increased in response to blue light stimulation (Fig. 1j, k). Putative unit waveform subtypes did not exhibit any significant differences in the probability of spiking in response to single low-frequency light pulses (Kruskal–Wallis = 0.76, *p* = 0.68) or 20 Hz pulse trains (Kruskal–Wallis = 1.51, *p* = 0.47), indicating that photostimulation recruited a range of DRN 5-HT neuron subtypes with differing intrinsic electrophysiological properties (Supplementary Figure 8). We did however note that putative units in the intact DRN never fired at or close to 20 Hz in vivo (Supplementary Figure 8c), despite the fact that patched 5-HT cells in acute midbrain slices remained faithful to the train up to 20 Hz (Fig. 1h). While over-clustering could lead to an underestimation of the individual unit firing rates, even the MUA recorded at the most active electrodes in the DRN in vivo did not achieve rates of 20 Hz during light train stimulation. This profound contrast between in vitro and in vivo fidelity at the single neuron level may be the result of local or distal feedback regulation within the intact DRN 5-HT circuit. The functional role of the different 5-HT neuron subtypes identified, in particular how they elicit different circuit elements, remains to be elucidated.

**Fig. 2:**
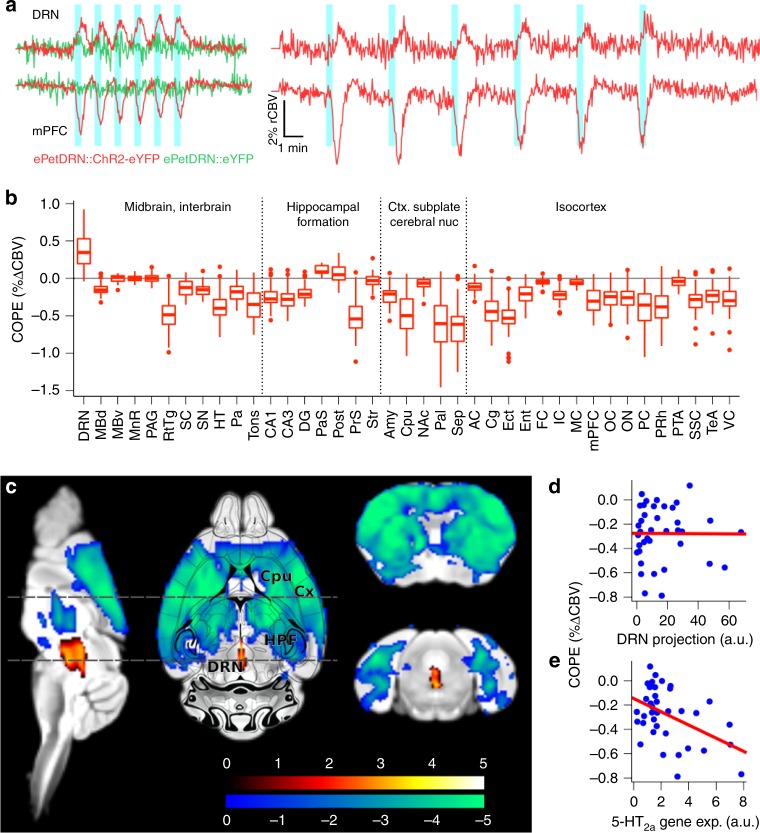
A whole-brain functional map of the DRN 5-HT circuit. **a** CBV traces from the DRN and medial prefrontal cortex (mPFC) during block stimulation with blue light (20 Hz, 5 ms pulse width, 6 × 20 s trains) during the short (*ePet*^*DRN*^*::ChR2-eYFP*, *n* = 10, *ePet*^*DRN*^*::eYFP*, *n* = 4) and long (*ePet*^*DRN*^*::ChR2-eYFP*, *n* = 7) fMRI protocols. **b** Contrast of parameter estimates (COPEs) extracted across 39 ROIs from *ePet*^*DRN*^*::ChR2-eYFP* using the short protocol (*n* = 10). They denote the relative response amplitude across the brain. Bound of box and centre line represent 25th, 50th, 75th percentiles, whiskers represent 1.5*inter-quartile range. **c** Second level voxel-wise analysis of *ePet*^*DRN*^*::ChR2-eYFP* (*n* = 10) against *ePet*^*DRN*^*::eYFP* (*n* = 4). Cx cortex, Cpu caudate-putamen, HPF hippocampal formation, DRN dorsal raphe nucleus. Colour scale = *t*-statistic (non-parametric test, *p* ≤ 0.05, cluster corrected). **d**, **e** COPEs extracted from 38 ROIs compared with **d** DRN projection field estimated with viral tracer and **e** 5-HT_2A_ gene expression estimated with in-situ hybridization, both obtained from AIBS database

To measure brain-wide responses to DRN serotonergic photoactivation, we recorded changes in cerebral blood volume (CBV) during optogenetic stimulation (ofMRI) using a block protocol consisting of 6 × 20 s, 20 Hz stimulus trains delivered either every 1 min or every 3 min (Fig. 2a). The acquired images presented minimal motion (mean frame-wise displacement 0.021 ± 0.008 mm) and geometric distortions, despite the implant (Supplementary Figure 9a–c). The method was preferred over blood oxygenation level dependent contrast (BOLD) due to enhanced detection power, suppression of large vessel signal and reduced susceptibility artefact (Supplementary Figure 9a, f, g)^7^. Importantly, CBV contrast leads to a seven-fold increase in response amplitude relative to that recorded with BOLD. In both stimulus paradigms, we found that CBV increased within the DRN during illumination, while projection areas including the medial prefrontal cortex predominately exhibited a tightly stimulus-locked CBV decrease, followed by an immediate return to baseline and rebound overshoot. Interestingly, the amplitude of the response decreased over the course of the six stimulation blocks in the projection areas, from 2.6% to 1.7%, but not in the DRN, suggesting an adaptation to enhanced 5-HT release within the DRN or projection areas. The shorter inter-block protocol was adopted to allow the acquisition of several scans within a single scan session. The CBV response was fitted with a general linear model (GLM) to facilitate unbiased voxel-wise comparisons between conditions across the entire brain. Contrast of parameter estimates (COPEs), representing the regional response amplitude, were estimated from every voxel using a gamma function model (Fig. 2b, Supplementary Figure 9d). A second-level comparison across the whole brain between *ePet*^*DRN*^*::ChR2-eYFP* (*n* = 10) and *ePet*^*DRN*^*::eYFP* (*n* = 4) revealed the extent of the elicited response, including a positive response confined within the DRN, and a negative CBV response distributed among the hippocampal formation, cortical subplate and striatum, and isocortex (Fig. 2d). Illumination in the absence of ChR2 produced no significant response, ruling out the possibility of light-induced heating artefacts (Fig. 2a, Supplementary Figure 9e). Interestingly, there was spatial overlap regarding the distal CBV response elicited with optogenetic stimulation with that evoked with chemogenetic stimulation of the 5-HT DRN neurons^8^, though the directionality of the response was opposed, and only optogenetically-induced responses matched the directionality of acute pharmacological stimulation of the DRN with a SSRI^8,9^. This severe discrepancy between two recently emerged stimulation methods, often discussed interchangeably in neuroscience research, exemplifies the potential extreme non-linear effects induced with acute and selective circuit manipulations^10^. Indeed, the activity evoked departs from basal physiological 5-HT firing patterns, which may explain this discrepancy. This highlights the relevance of intact whole-circuit visualisation in support of behavioural observations or other modalities.

### DRN photoactivation suppresses cortical neuronal activity

**Fig. 3:**
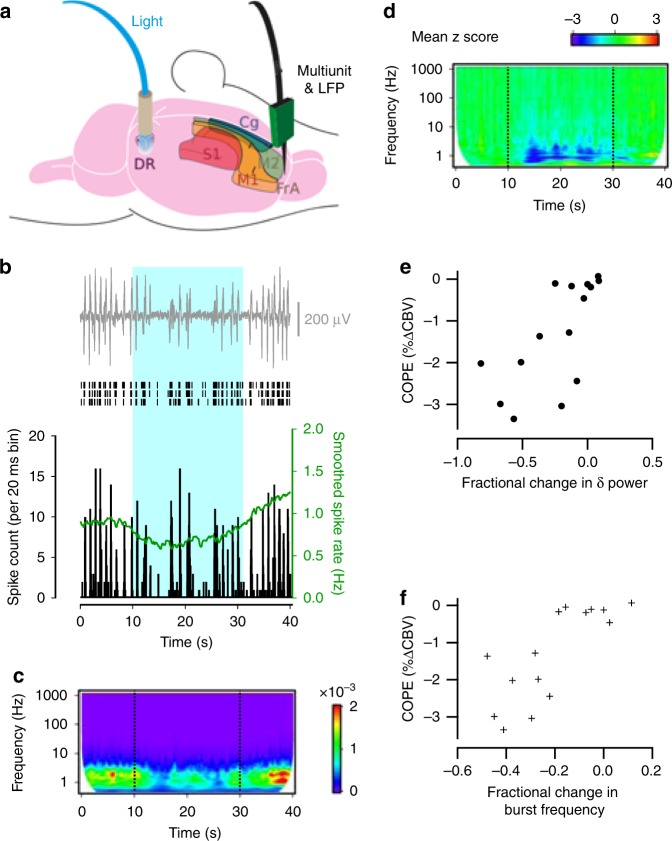
Photoactivation of DRN 5-HT neurons dampens MUA and LFP power in cortical projection areas. **a** Schematic of the experimental set-up for paired MUA and LFP recordings in the DRN and projection areas during photostimulation. **b** Cortical LFP (top), raster plots (middle) of spikes recorded on 3 neighbouring channels and spike time histogram (bottom), reveal reduction in the frequency of cortical network bursts and MUA during optogenetic activation of DRN 5-HT neurons. **c** Wavelet amplitude spectrum of the cortical LFP averaged over 6 stimulation trials for the experiment shown in (**b**) (warmer colours indicate higher power). Dashed lines indicate onset and end of photostimulation. **d** Z transform of the wavelet amplitude spectrum averaged across recording sites of mice with robust CBV response to photostimulation, showing a consistent reduction in the amplitude of low-frequency LFP components (8 recording sites from 3 mice). **e**, **f** COPEs from various cortical regions correlate strongly with fractional changes in both the **d** delta power (Pearson *r* = 0.69; *p* = 0.004) and **e** burst frequency (Pearson *r* = 0.78; *p* = 0.0006) recorded in 15 recording sites from 7 mice

For fMRI responses in the monkey visual cortex elicited by rotating polar-transformed chequerboard patterns, a cognitive process mediated by glutamatergic and GABAergic signalling amongst networks of pyramidal neurons and interneurons, local field potentials (LFPs) were found to better estimate the BOLD response compared to MUA^11^. This finding suggested that fMRI hemodynamic signals reflect the input to, and intracortical processing within, a specific area rather than its spiking output^11^. However, it remains unclear whether this observation can be generalised to other forms of neuronal communication such as 5-HT mediated signalling. We therefore performed intracranial recordings from a variety of cortical regions and compared LFP and spiking behaviour with the hemodynamic response to transient photoactivation of DRN 5-HT neurons (Fig. 3a).

Similarly to the glutamatergic response to a behavioural stimulus^11^, the 5-HT response to photoactivation represents synaptic signal integration. Specifically, synchronous with photoactivation of DRN 5-HT neurons, cells in the frontal, somatosensory, motor and anterior cingulate cortex exhibited stimulus-evoked decreases in both MUA and LFP power that rapidly returned to baseline upon stimulus termination (Fig. 3b). Wavelet analysis revealed that delta bands were most affected, gradually declining in power upon optogenetically-triggered 5-HT release in the cortex and returning to baseline upon termination of the stimulus train (Fig. 3c, d). Changes to LFP power occurred following a post-stimulus delay of about 5 s. We also observed that the fractional cortical delta power (Fig. 3e) and the fractional change in burst frequency (Fig. 3f) were both inversely correlated with between-subject and regional variance in response amplitude measured in the same group of animals during ofMRI. The convolved LFP-derived band power correlated markedly with the CBV response evoked by 5-HT release (*r*_delta_ = 0.75 ± 0.16, *r*_gamma_ = 0.24 ± 0.6, Supplementary Figure 10), while no discernible correlations could be established between convolved MUA signal and CBV (*r*_MUA_ = -0.1 ± 0.34) for animal- and region of interest (ROI)-matched recordings.

### ofMRI response is independent of projection density

We next investigated cellular and molecular factors determining functional connectivity of the DRN 5-HT circuit by comparing the whole-brain DRN/5-HT hemodynamic maps derived from ofMRI with the DRN/5-HT neuron projection density maps from the Allen Institute for Brain Science (AIBS). Prior to this comparison, we removed white matter structures from the projection maps to ensure that our analysis focused on 5-HT terminals rather than fibre tracts (Supplementary Figure 11). Surprisingly, we did not find significant correlations (Fig. 2d, Supplementary Figure 12a) indicating that the anatomical architecture of the central 5-HT circuitry as such does not determine the functional consequences of its activation. These results are consistent with the finding that functional connectivity originating from photoactivated dopamine neurons of the ventral tegmental area (VTA) does not correlate well with VTA dopaminergic neuron projection density^12^, but are in stark contrast to recent resting-state fMRI observations that indicate intracortical functional connectivity relationships at a macroscopic level correlate with intracortical structural connectivity^13–15^.

We therefore examined another possible determinant for regional-variance of hemodynamic responses elicited by DRN 5-HT photostimulation, namely the specific expression patterns of 5-HT receptors. The 5-HT receptor family is comprised of both metabotropic and ionotropic sub-types that can be either excitatory or inhibitory, depending on downstream signalling cascades and cellular localisation^1^, and may strongly influence the net effect of 5-HT release on functional connectivity. We explored this possibility using a series of additional brain-wide voxel correlation maps from the best available 5-HT receptor subtype expression data currently available from the AIBS. Of the 14 5-HT receptor subtypes listed in the database, we judged 5 expression maps to be sufficiently robust for our analysis (Supplementary Figure 11); the remaining AIBS maps either containing strong artefacts or no signal. Significant correlations emerged between the patterns of 5-HT_1F_, 5-HT_2A_ and 5-HT_2C_ receptor density and the hemodynamic response, while distribution of 5-HT_1A_ and 5-HT_1B_ receptors did not match the ofMRI response pattern, similar to the comparison with DRN 5-HT projections (Fig. 2e, Supplementary Figure 12b–f). Since we were unable to assess all 5-HT receptor subtypes, we cannot conclude whether these first three receptor subtypes determine functional connectivity of the DRN 5-HT circuit; nevertheless, 5-HT_1F/2A/2C_ explain 52.6%, 21.2% and 30.8% of the variance in the hemodynamic response elicited by DRN 5-HT photostimulation, respectively. Interestingly, 5-HT_1A_ and 5-HT_1B_ receptors are autoreceptors expressed either somatodendritically on 5-HT neurons of the DRN or presynaptically within axon terminals of DRN 5-HT neurons^16,17^, while 5-HT_1F_, 5-HT_2A_ and 5-HT_2C_ receptors are all expressed post-synaptically^18,19^. We therefore conclude that post-synaptic receptor density, compared to neuroarchitecture, is the rather surprising dominant factor underlying DRN 5-HT functional connectivity following our photoactivation protocol. All remaining variance is likely governed by indirect poly-synaptic connections, an expected contribution given other recent fMRI findings^15^. This finding may hold for other neuromodulatory systems including dopamine and neuropeptides as well, for which projection density can be surprisingly sparse in regions that exhibit high levels of receptor expression. Ultimately, the emerging data depict a potential level of macro plasticity in neuromodulatory circuits that could not be attained if neuroarchitecture dominated neuroconnectivitiy, as has previously been assumed^20^.

### Acute stress occludes the ofMRI response

**Fig. 4:**
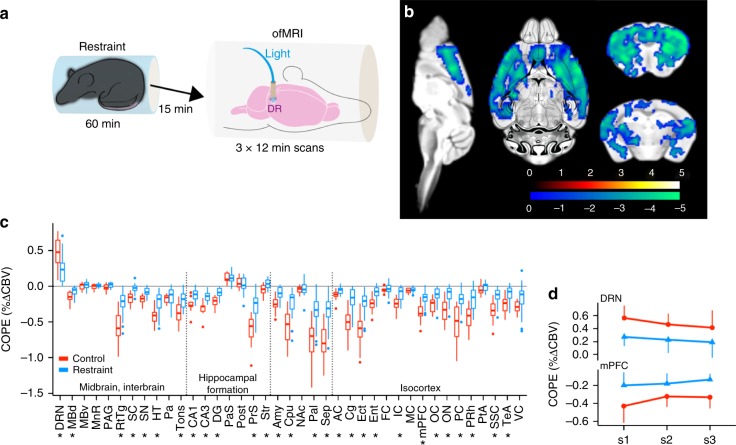
Acute stress occludes photoactivation of the DRN 5-HT circuit. **a** Experiment schematic. **b** Second level analysis comparing restraint (*n* = 7) to control (*n* = 4) condition indicate a decrease in negative response amplitude in the restraint group relative to control across the brain. Colour scale = *t*-statistic (non-parametric test, *p* ≤ 0.05, cluster corrected), blue indicating significance for restraint > control comparison; see Supplementary Figure 13 for all brain slices. **c** COPEs comparing restraint (blue) and control (red) indicate reduced response amplitudes in the majority of responding ROIs, including DRN (*p < 0.05, FDR corrected). Bound of box and centre line represent 25th, 50th, 75th percentiles, whiskers represent 1.5*inter-quartile range. **d** The blunted response to photoactivation following acute stress persists across the three consecutive scans of each session. Error bars indicate ±1 standard deviation

The DRN represents an important element of the acute stress response^21,22^, exemplified by activation of 5-HT neurons and an increase in the synaptic availability of 5-HT throughout much of the brain following forced restraint^23,24^. To determine how functional circuit changes might be affected by acute stress, we subjected 7 *ePet*^*DRN*^*::ChR2-eYFP* mice to a brief immobilisation period immediately prior to ofMRI and compared the results to 4 *ePet*^*DRN*^*::ChR2-eYFP* stress-free controls (Fig. 4a). We found a marked reduction in the hemodynamic response to DRN 5-HT photoactivation in acutely-stressed animals versus controls (Fig. 4b, c; Supplementary Figure 13). Moreover, this effect persisted throughout three successive ofMRI scans (Fig. 4d), indicating that the consequences for DRN 5-HT function elicited by acute stress persisted throughout the duration of the experiment.

Since the hemodynamic responses were blunted by acute stress within DRN projection areas as well as the DRN itself, we normalised the COPEs of projection areas to the COPE of the DRN to determine whether the functional connectivity of the DRN 5-HT circuit was affected. No statistical difference between acutely restrained animals and controls was apparent (Supplementary Figure 14), indicating that following acute stress, functional connectivity per se is actually not affected.

We presume the global blunting effects of stress on photoactivation represent a ceiling effect since stress can lead to DRN sensitisation accompanied by sustained increases in 5-HT release for up to 24 h^25–27^. Such a scenario would produce less room for further inhibition, attenuating the hemodynamic response to DRN 5-HT neuron photoactivation, which is what we observed here. The change in response amplitude following restraint highlights the potential of ofMRI for investigating neurological implications of salient life experiences. The amplitude of photoactivation-elicited responses may serve as a measurable and objective proxy for stress, enabling detailed examination of procedures that might support stress resilience, such as anxiolytic agents and behavioural enrichment.

### Circuit regulation of DRN 5-HT connectivity by fluoxetine

**Fig. 5:**
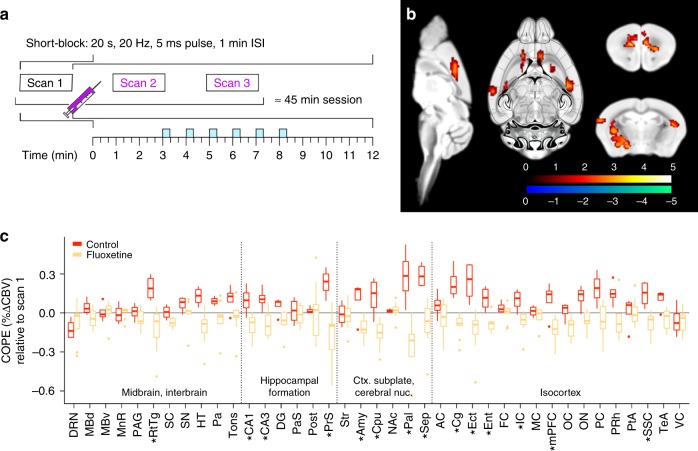
Circuit regulation of DRN 5-HT functional connectivity by fluoxetine. **a** Experiment schematic. **b** Second level analysis comparing fluoxetine (*n* = 9) to control (*n* = 4) condition indicate an increase in negative response amplitude in scan 2 and 3 relative to scan 1 in the prefrontal, cingulate and peri-hippocampal cortex, as well as amygdala and parts of the striatum, in the fluoxetine group. Colour scale = *t*-statistic (non-parametric test, *p* ≤ 0.05, cluster corrected), red-yellow colour bar indicating significance for fluoxetine < control comparison; see Supplementary Figure 15 for all brain slices. **c** COPEs comparing fluoxetine (yellow) and control (red) confirm an augmentation of the decreased response amplitudes in CBV within regions highlighted by the voxel-wise analysis (*p < 0.05, FDR corrected). Bound of box and centre line represent 25th, 50th, 75th percentiles, whiskers represent 1.5*inter-quartile range

As the primary source of 5-HT for the CNS, the DRN is presumed critical for SSRIs mode of action. However, the mechanism underlying the effect of SSRIs on the intact DRN 5-HT circuit remains unclear beyond the knowledge that fluoxetine, like acute stress, elevates synaptic availability of 5-HT^28^. We therefore used ofMRI to study SSRIs by administering a pharmacologically relevant dose^29^ of fluoxetine via tail vein infusion during ofMRI in 9 *ePet*^*DRN*^*::ChR2-eYFP* mice and compared the results to 4 *ePet*^*DRN*^*::ChR2-eYFP* non-injected controls (Fig. 5a). Fluoxetine administration elicited greater response amplitude in the prefrontal and cingulate cortex, as well as amygdala and striatum (Fig. 5b, c, Supplementary Figure 15). The ability to monitor animals non-invasively with ofMRI is thus expected to reveal the circuit re-organisation taking place longitudinally and shed light on currently obscure mechanisms of anti-depressant action.

## Discussion

The 5-HT system constitutes the most diverse signalling network of the CNS and major physiological elements of its circuit characteristics remain unclear. Here we demonstrate the possibility to visualise the whole circuit in the intact mammalian brain in conjunction with psychiatric-relevant stimuli. Several surprising findings emerged from this analysis. First, we observe that photostimulation of the DRN within *ePet*^*DRN*^*::ChR2-eYFP* mice recruits a variety of 5-HT neuronal subtypes distinguished by their waveforms. Second, this stimulation leads to an increase in CBV within the DRN that coincides with decreases in CBV throughout much of the brain, demonstrating that despite the expression of excitatory and inhibitory 5-HT receptors, the net effect of 5-HT release in most areas is inhibitory. Third, the hemodynamic response to photostimulation correlates with LFP delta oscillations and, like glutamatergic-induced hemodynamic responses, likely corresponds to synaptic integration rather than neuronal spiking. Fourth, functional connectivity of the DRN 5-HT network is rather remarkably uncoupled from its underlying neuroanatomical architecture, and instead primarily governed by the far more plastic regional differences in the expression profiles of post-synaptically expressed 5-HT receptors. Fifth, acute stress shunts further activation of the DRN 5-HT circuit without affecting absolute functional connectivity. Sixth, fluoxetine exerts the opposite effect as acute stress by enhancing DRN 5-HT-induced suppression of brain activity within restricted regions of the central 5-HT circuit.

These results help understand basic principles of the DRN 5-HT circuit, providing novel insights on the neural correlates underlying the hemodynamic responses that follow DRN 5-HT neuron activation. The widespread inhibition observed here contrasts with the subtle behaviour response mediated with 5-HT DRN photostimulation, e.g. inhibition of spontaneous locomotor activity^30^, inhibition of somatosensory activity^31^, despite comparable paradigms. This apparent discrepancy between visualized neuronal activity and behavioural outcome should be explored in greater depth. For instance, ofMRI would provide a crucial tool to explain behavioural variability in a population of animals. Additionally, these results provide a potential neurophysiological explanation for the divergent outcomes triggered by acute stress and acute fluoxetine administration, despite the fact that both of these acute stimuli increase 5-HT synaptic availability. The most important discovery however may be that post-synaptic receptor expression density, and not DRN 5-HT neuroarchitecture, is the primary determinant of functional connectivity within the central 5-HT system. Our study therefore highlights the existence of a far more extended functional connectome relative to what structural connectivity of the DRN alone would indicate.

## Methods

### Experimental subjects

All experiments and manipulations conformed to the guidelines set by the Animal Care Commission of Switzerland and were covered under the authority of animal permit ZH263/14 belonging to B.J.S. and in accordance with the UK Animals (Scientific Procedures) Act 1986. All possible measures were taken to ensure minimal pain and discomfort. *B6.Cg-Tg(Fev-cre)1Esd/J* (ePet-cre mice; RRID:IMSR_JAX:012712) males and females, 8–16 weeks of age, were used in this study. *ePet-cre* Genotyping was complete using forward primer AAAATTTGCCTGCATTACCG, reverse primer ATTCTCCCACCGTCACG and an annealing temperature of 57 °C.

### Histology

Fluorescent imaging: Animals were anesthetized with a lethal cocktail of ketamine (120–150 mg kg^−1^) and medetomidine (0.5–1.0 mg kg^−1^) administered i.p. and then perfused with 10–15 ml ice-cold PBS followed by 10–15 ml ice-cold 4% paraformaldehyde (PFA) in phosphate buffered saline (PBS). The whole brain was then removed and stored in 4% PFA in PBS at 4 °C for at least 24 h. Brains were sectioned on a vibratome (Leica, Germany), permeabilized with 0.1% Triton-X in PBS for 10 min, processed according to target of interest (Supplementary Table 1) and mounted in VectaShield medium (Vector Laboratories, CA, USA) according to manufacturer instructions. Fluorescence was captured using a Leica DFC365FX camera mounted on a Leica M165F6 wide-field fluorescent stereoscope (Leica, Germany), or confocal microscope (Leica SP8, or Olympus Fluoview 1000).

### Surgical procedures

Virus delivery: The surgery area and equipment were sterilized with 70% ethanol, a bead sterilizer and/or autoclaving where possible. Subjects were anesthetized with 3% isoflurane in an anaesthetic 12 cm^3^ chamber. Once fully anesthetized, subjects were weighed and transferred to a stereotaxic apparatus by gently fixing the head with ear bars and softly clamping the open snout on a nose piece that provided continuous isoflurane as anaesthetic blended with oxygen and air to a minimum of 30% oxygen. Throughout surgery, subjects overlaid a feedback-controlled heating pad receiving information from a lubricated rectal probe to assess core temperature (maintained between 35 and 37 °C). Subject breathing was continually monitored and anaesthesia regulated accordingly. The subject’s eyes were protected with Vaseline or vitamin A tear gel. Betadine ointment as aseptic and lidocaine/prilocaine as a topical analgesic (EMLA cream) were applied topically to the precise incision area on the scalp. An s.c. injection of Meloxicam (Metacam) analgesic was given in sterile saline (0.5 mg ml^−1^; 5 µl g^−1^) using a 30G needle. After testing for analgesia by gentle tail and/or hind paw pinch, a sharp scalpel was used to expose the skull. All membranes were pushed aside and the skull surface cleaned with mild hydrogen peroxide (not exceeding 10%) to remove remaining membranes and bleach the connective tissue, enabling clear visualization of cranial reference points. A remote, pedal-driven drill affixed to the stereotaxic manipulator was next used to create a 400 µm diameter craniotomy at coordinates −0.6 mm from Lamda, 1.0 mm from midline. A stainless steel 33G infusion cannula (Plastics One, WV, USA) affixed to the manipulator and connected to a 50 µl gas-tight syringe (Hamilton, Switzerland) via infusion tubing (Plastics One, WV, USA) and loaded with AAV packaged with *EF1a.DIO.hChR2(H134R)-eYFP.WPRE.hGH* (AV-1-20298P; Penn Vector Core, PA, USA) was then lowered at 20° off the normal axis, 3.6 mm beyond the brain dura. Infusion of 1.0 µl AAV ensued over the course of 10 min (0.1 µl min^−1^). An additional 5 min were then allowed for diffusion before the infusion cannula was gradually removed. Finally, the skin was pulled over the skull and sutured with a sterile curved needed and non-absorbable sutures. Betadine and EMLA cream were again applied to the now closed surgery area and the subject was removed from the apparatus, weighed and placed in a clean and heated recovery chamber with close monitoring until behaving normally. The subjects were then returned to their home cage (group housed) and monitored at least once a day for 3 days. If any measure from the postoperative monitoring sheet received a score greater than 1, an additional dose of general analgesic (Metacam in sterile saline; 0.5 mg ml^−1^; 5 µl g^−1^; s.c.) was administered. Non-absorbable sutures typically grew out from the skin within 2 weeks, and if not, were removed during subsequent optical cannula implant.

Optical implants: Implantation of fMRI-friendly optical fibre cannulae occurred 1–2 weeks post-viral infusion and at least 1 week prior to ofMRI. Preparation of the surgery area and initial surgery steps proceeded identically to viral infusions (see ‘virus delivery’ above). Once the skull was exposed and membranes cleared, a 600 µm hole was drilled into the position for fibre implantation, directly on midline at Lamda −0.6 mm. A 400 µm optic fibre extending 3.3 mm beyond the fibre casing obtained from Doric Lenses (Quebec, Canada) was gently lowered until the casing became flush with the skull. Any bleeding was cleaned away with the finely-twisted end of a sterile cotton swab. The skull was then re-hydrated by applying PBS with a cotton swap. After providing about 30 s for the skull to hydrate, a layer of etching reagent (iBond Total Etch, Heraeus Kulzer, Germany) was applied to the skull using the manufacturer’s accompanying applicator. After 30 s, another layer of etching reagent was applied and then fixed with 15 s illumination with a 6 mW blue LED (Elipar S10, 3M, Switzerland). Optic cannulae were then cemented in place using light-curing dental cement (Tetric EvoFlow, Ivoclar Vivadent, NY, USA), providing a minimum of 3 mm of unobstructed cannula above the cement layer to enable coupling to the optic fibre. Finally, the cannula holder was raised away from the fibre and the skin fixed to the base of the cement with veterinary tissue glue (Surgibond, Eisenhut-Vet, Switzerland). Finally, animals were allowed to recover in an identical manner reported above (see ‘virus delivery’).

Craniotomies for acute electrophysiological recordings in vivo: Subjects containing optic implants targeting the DRN were anesthetized using 3% isoflurane in a 30%-minimum oxygen/air blend, and transferred to a mouse stereotaxic frame providing continuous circulating isoflurane at roughly 2% in the same gas vehicle mixture as required according to the animal’s breathing. To provide access of a recording electrode to 5-HT neurons of the DRN, dental cement was progressively removed using a foot-powered drill on the right side of the optical cannula until the skull was exposed. For subjects in which dual recordings were to be made, the cement overlaying the projection ROI was also removed in an identical manner. Craniotomies were performed by removing the skull overlaying the cortex lateral to the DRN using the drill at low speed to gently grind off successive layers of a circle encasing the desired region. Once the skull at the circle’s edge was completely removed, fine tip forceps were used to lift away the remaining plate of cortex, and the dura was punctured and removed with the same tool. Finally, anti-coagulate sponge fully-hydrated with room temperature PBS was added over the exposed brain. Once all craniotomies were complete, a bolus of medetomidine (0.1 mg kg^−1^) was delivered s.c., and after 5 min, the concentration of isoflurane was reduced to 0.5% and medetomidine was continuously delivered at (0.2 mg kg^−1^ per hour) s.c., in a manner identical to the ofMRI experiments.

### Electrophysiology

Patch-clamp recordings in vitro: Mice were decapitated under isoflurane anaesthesia, and the brains removed in ice-cold oxygenated cutting solution, containing (in mM): N-methyl d-glucamine (135), KCl (1), CaCl_2_ (0.5), MgCl_2_ (1.5), KH_2_PO_4_ (1.2), choline bicarbonate (20), D-glucose (10), with pH adjusted to 7.4 with HCl (resulting in a final [Cl-] of ~145 mM). Coronal slices (350 μm) were prepared using a Vibratome VT1200S (Leica, Germany), transferred to an interface recovery chamber filled with artificial cerebrospinal fluid (aCSF) containing (in mM): 126 NaCl, 3 KCl, 1.25 NaH_2_PO_4_, 1.2 MgSO_4_, 1 CaCl_2_, 26 NaHCO_3_ and 10 glucose, with pH 7.2–7.4 when bubbled with carbogen gas (95% O_2_ and 5% CO_2_). The slices were maintained at 32–34 °C for at least 30 min, before being allowed to cool to room temperature. For recordings, slices were transferred to a submerged chamber, and superfused with carbogenated aCSF heated to 32–34 °C at 2–4 ml min^−1^. Neurons were visualized under infrared oblique illumination (Olympus, BX51WI, 40× water-immersion objective). Whole-cell current-clamp recordings were performed with glass pipettes (5–8 MΩ), pulled from standard borosilicate glass, and filled with a pipette solution containing (in mM): 110 potassium-gluconate, 40 HEPES, 2 ATP-Mg, 0.3 GTP, 4 NaCl and 4% biocytin (wt/vol) (pH 7.2–7.3; osmolarity 280–290 mosmol l^−1^). Recordings were acquired using a Multiclamp 700B amplifier (Molecular Devices), and digitised using an ITC-18 A/D board (Instrutech). Blue light was delivered via a galvanometer-based movable spot illumination system coupled to the epifluorecscence port of the microscope using a single mode fibre (473 nm, 5–25 ms, UGA-40, Rapp OptoElectronic). Stimulation and recordings were controlled via custom-written procedures in Igor Pro (Wavemetrics). Isoflurane was dissolved in an air-tight container of aCSF using conditions previously shown to induce a final concentration comparable to 1 MAC for C57Bl/6 mice.

Multielectrode recordings and optogenetic activation in vivo: Recordings were performed using single-shank 16 site silicon probes, with electrode spacings of 25 µm, 100 µm (Neuronexus Technologies Inc., MI, USA) or 200 µm (Cambridge NeuroTech, UK). Recordings from the dorsal raphe were performed with 25/100 µm spaced electrodes, with recordings from target structures performed using 100/200 µm spaced electrodes. Each shank was gently lowered progressively to the desired coordinates (Supplementary Table 2). Recordings from multielectrode arrays were performed using Brainware (Tucker Davis Technologies, Alachua, FL, USA), with traces for detecting multiunit activity band-pass filtered between 0.3 and 3 kHz and digitised at 25 kHz, and traces for LFP recordings low-pass filtered at 1.9 kHz, digitised at 25 kHz, and down-sampled by a factor of 8 for file storage. The source of blue light was a 473 nm laser (Thorlabs, Germany; selected for ease of transport) used to deliver 4–40 mW of power (Fig. 1I). Laser power was controlled with the bench-top unit, and verified with a light meter (PM 160, Thorlabs, Germany). Pulse duration, inter-stimulus and inter-train intervals were controlled with in-house software designed in LabView (National Instruments, Switzerland). At the end of the recording session, the animal was overdosed with sodium pentobarbitone and perfused with 10–15 ml ice-cold PBS followed by 10–15 ml ice-cold 4% PFA in PBS.

Analysis of electrophysiological data: Data were analysed using custom-written procedures in Igor Pro (Wavemetrics). Extracellular spikes were detected as signals exceeding 5 standard deviations of the noise. For recordings from the dorsal raphe using 25 µm spaced linear probes, an adapted spike sorting procedure^32^ was used to explore whether neurons displaying specific spike waveforms were selectively recruited by optogenetic stimulation. Briefly, spike metrics were converted into *z* scores, over-clustered using an in-built *k* means algorithm, and progressively aggregated if the intercluster distance was <2.5 and merging did not produce violations of refractory period of 2 ms. Analysis was first performed for spikes which could be observed on 2 channels (stereotrode data), and subsequently on the residual single channel spikes, with auto-correlation and cross-correlation plots used to validate the clustering procedure. As several waveform clusters appeared to exhibit rapid adaptation during optical trains, clusters containing >50 spikes were included for subsequent analysis. Spike metrics from the average waveform for each cluster were used to identify different waveform types via a *k* means algorithm. This clustering procedure is likely to be conservative, and underestimate the firing rate of individual neurons, but was deemed sufficiently robust to detect any bias in optogenetic recruitment.

Significant differences in spiking behaviour were examined with a Kruskal–Wallis test followed by Dunn’s post-hoc comparison test. Statistics are reported for combined analysis of stereo and single channel clusters, but the same pattern of statistical significance was also observed for stereo clusters alone.

### Functional magnetic resonance imaging

Animal preparation: Animals were anesthetized with isoflurane (induction 3%, preparation 2%) in a 20/80% O_2_/air mixture. Animals were positioned on a MRI-compatible cradle equipped with a face mask, rectal thermometer and adjustable warm water flowing within the support. Animal temperature was kept at 36.5  ± 0.5 °C throughout the experiment. A cannulae was placed in the tail vein to administer agents. A s.c. line was placed on the animal flank to administer complementary anaesthetic (Dormitor, medetomidine hydrochloride; Pfizer Pharmaceuticals, UK). After animal positioning, a bolus of medetomidine was injected s.c. at 0.1 mg kg^−1^. After 5 min post-bolus, isoflurane was reduced to 0.5% at the initiation of continuous infusion of medetomidine was initiated (0.2 mg kg^−1^ per hour) to maintain the sedation for the remainder of the scanning session. For CBV fMRI experiments, the paramagnetic iron oxide nanoparticle-based intravascular contrast agent Endorem® (Laboratoire Guerbet SA, France) was injected at a dose of 30 mg kg^−1^ Fe, and given 10 min to reach steady-state prior to imaging.

MRI: Functional MRI was performed on a 7 T Pharmascan scanner (Bruker BioSpin MRI, Ettlingen, Germany), operating at 300 MHz. A custom-built transmit-receive surface coil was positioned on the head of the animal. A light fibre connected to a laser (LuxX® 488-60, Omicron, Germany) was positioned through the coil and attached to the zirconia fibre insert on the mouse head with a zirconia sleeve. Images acquisition was performed with Paravision 6 software. High-resolution anatomical images were acquired using a gradient echo FLASH sequence to serve as references with repetition time (TR) 1500 ms, echo time (TE) 1.97 ms, flip angle (FA) 50°, matrix size (MS) 120 × 120, field of view 20 × 17.5 mm, slice thickness 0.5 mm, slice gap 0.15 mm, 14 slices. CBV fMRI was acquired with multi-shot gradient echo EPI using the same geometry as the anatomical image, 2 segments, TR 1000 ms. TE 5.6 ms, FA 90°, MS 64 × 64, bandwidth 250,000 Hz, 360 or 720 repetitions for a total duration of 12 and 24 min, corresponding to the short-block (Fig. 2a) and long-block protocol, respectively. The echo time was changed to 15 ms for BOLD fMRI. Correction for magnetic field inhomogeneity was performed with Mapshim using an ellipsoid ROI covering the whole brain. A trigger device was used to control laser onset with respect to the fMRI scan. Laser power was controlled via the accompanying Omicron software. Laser stimulation was performed with 6 blocks of 20 s ON and 40 s OFF for 12 min scans (short-block protocol), and 20 s ON and 160 s OFF for 24 min scans (long-block protocol), and controlled via an in-house LabView program (National Instruments, Switzerland). Conditions: Short CBV scans (12 min) were performed in a series of 3 per session: (a) 3 scans with laser power set at 100%, used as a control group for the subsequent analysis, (b) 3 scans with laser power set at 100%, 66% and 33% in varying order, (c) 1 baseline scan, i.v. administration of Fluoxetine (4.5 mg kg^−1^ ^29^), 2 scans post Fluoxetine, (d) 60 min pre-scan animal restrain, 3 scans post restrain.

Data processing: Data processing was performed with FSL (5.0.8, https://fsl.fmrib.ox.ac.uk/) and AFNI (2011_12_21_1014, https://afni.nimh.nih.gov/) and BROCCOLI (2015-09-11, https://github.com/wanderine/BROCCOLI)^33^. Anatomical images from each scan session were linearly aligned with one another, flipped and merged to generate a symmetrical reference template. Linear and non-linear transformations were estimated between the anatomical images and the reference template using FLIRT and FNIRT. Functional images were temporally realigned (3dVolreg), the linear and non-linear transformations from the anatomical images were then applied to the functional images. The temporal signal for each region was extracted from a set of ROIs based on the reference anatomical images. The time series were linearly detrended to account for the iron nanoparticle clearance, normalised as percent change to baseline, and the sign inverted. For voxel-wise analysis, the functional images were smoothed with a 0.45 mm^2^ kernel (3dBlurtoFWHM). A GLM first-level analysis was applied to each scan individually using BROCCOLI. The parameters of the response to the stimulation blocks were modelled into separate regressors using the default hemodynamic response function convolution together with temporal derivatives for the activity regressors and polynomial detrending regressors to account for linear and non-linear drifts. A contrast was designed to obtain COPE at every voxel. Visual inspection of the residuals from the analysis for each scan suggested the model accounted fully for the response in the time series for each condition.

Statistical analysis: Second-level between-group voxel-wise statistics was carried using non-parametric permutation testing implemented in BROCCOLI. A design matrix modelling scan order for each condition (control, fluoxetine and restraint) and gender as a covariate was used for the analysis. Fluoxetine versus control comparisons were tested by using a within-session correction to account for the inter-animal variability. Contrasts were designed so that the two scans post-drug injection were averaged, and subtracted with the pre-drug scan and compared against the control scans processed similarly. For the restrain versus control comparison, within-session correction could not be applied; all 3 scans in the sessions were averaged and compared against the control scans. The null distributions for each comparison were estimated using 5000 permutations, and the estimated *p*-values were corrected using cluster extend correction. Corrected *t*-statistic maps are shown as overlay on the AMBMC template (Australian Mouse Brain Mapping Consortium, https://www.imaging.org.au/AMBMC). Statistical analysis across ROIs were corrected using false discovery rate (FDR). Descriptive statistics are given as mean ± 1 standard deviation.

### Reporting summary

Further information on experimental design is available in the Nature Research Reporting Summary linked to this article.

## Supplementary information

Supplementary Information

Reporting Summary

## Data Availability

Study data are available in BIDS format at the https://openneuro.org/DataRepository (Project_ID: Mouse_opto_DRN, 10.18112/openneuro.ds001541.v1.1.2).
