# Author Correction: A brain-wide functional map of the serotonergic responses to acute stress and fluoxetine

**DOI:** 10.1038/s41467-021-21192-6

**Published:** 2021-02-15

**Authors:** Joanes Grandjean, Alberto Corcoba, Martin C. Kahn, A. Louise Upton, Evan S. Deneris, Erich Seifritz, Fritjof Helmchen, Isabelle M. Mansuy, Edward O. Mann, Markus Rudin, Bechara J. Saab

**Affiliations:** 1 Singapore Bioimaging Consortium, Agency for Science Technology and Research, 11 Biopolis Way, Singapore, 138667 Singapore; 2 Neuroscience Center Zurich, Winterthurerstr. 190, CH-8057 Zurich, Switzerland; 3 Institute for Biomedical Engineering, University of Zurich and ETH Zurich, CH-8093 Zurich, Switzerland; 4 Center for Psychiatric Neuroscience, University of Lausanne, CH-1008 Prilly-Lausanne, Switzerland; 5 Preclinical Laboratory for Translational Research into Affective Disorders, Department of Psychiatry, Psychotherapy and Psychosomatics, University of Zurich Hospital for Psychiatry, August-ForelStr. 7, CH-8008 Zurich, Switzerland; 6 Department of Physiology, Anatomy and Genetics, University of Oxford, Oxford, OX1 3PT UK; 7 Oxford Ion Channel Initiative, University of Oxford, Oxford, OX1 3PT UK; 8 Department of Neurosciences, Case Western Reserve University School of Medicine, 10900 Euclid Ave., Cleveland, OH 44106-4975 USA; 9 Brain Research Institute, University of Zurich, Winterthurerstr. 190, CH-8057 Zurich, Switzerland; 10 Laboratory of Neuroepigenetics, Brain Research Institute, Medical Faculty of the University of Zürich, Institute for Neuroscience, Department of Health Science and Technology of ETH Zürich, Irchel Campus Room Y55H66, Winterthurerstrasse 190, CH-8057 Zürich, Switzerland; 11 Institute of Pharmacology and Toxicology, University of Zurich, CH-8093 Zurich, Switzerland; 12 Present Address: Mobio Interactive, Thurwiesenstrasse 4, CH-8037 Zurich, Switzerland

**Keywords:** Neuroscience, Neural circuits

Correction to: *Nature Communications* 10.1038/s41467-018-08256-w, published online 21 January 2019.

The original version of this Article omitted from the author list the 8th author Isabelle M. Mansuy who is from the ‘Laboratory of Neuroepigenetics, Brain Research Institute, Medical Faculty of the University of Zürich, Institute for Neuroscience, Department of Health Science and Technology of ETH Zürich, Irchel Campus Room Y55H66, Winterthurerstrasse 190, CH-8057, Zürich, Switzerland’.

Consequently, the following was added to the Acknowledgements: ‘and funding from University Zürich, ETH Zürich and the Swiss National Science Foundation (grant 31003A-135715) awarded to I.M.M.’ The following text was added to the “Methods” section ‘ZH150/11 given to I.M.M. and ‘. The following text was added to the Author Contributions: ‘B.J.S. and A.C. performed some experiments reported in this article while supervised by I.M.M’. This has been corrected in both the PDF and HTML versions of the Article.

B.J.S. disagrees with the addition of Isabelle Mansuy as an author.

