# Supplementary material for: A brain-wide functional map of the serotonergic responses to acute stress and fluoxetine
Source: Nat Commun. 2019 Jan 21;10:350. doi: 10.1038/s41467-018-08256-w (PMC6341094; doi:10.1038/s41467-018-08256-w)
Supplement: Supplementary file 1 — Supplementary Information [file 41467_2018_8256_MOESM1_ESM.pdf]

Supplementary information

A brain-wide functional map of the serotonergic responses to acute stress and fluoxetine

by *Joanes Grandjean et al.*

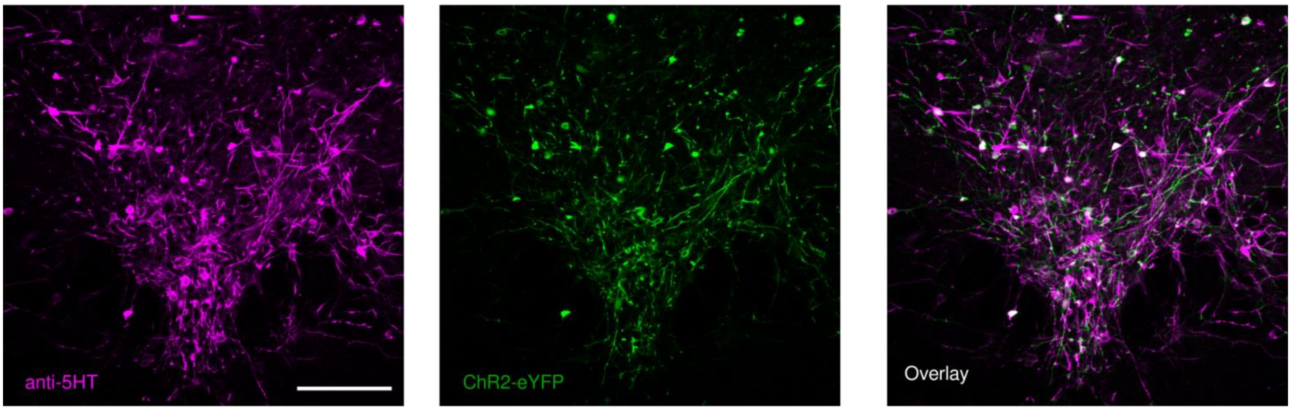

Supplementary Figure 1. Colocalisation of 5-HT (red) and ChR2-eYFP (green) in cell bodies (yellow) within the dorsal raphe nucleus. Scale bar indicates 500 µm.

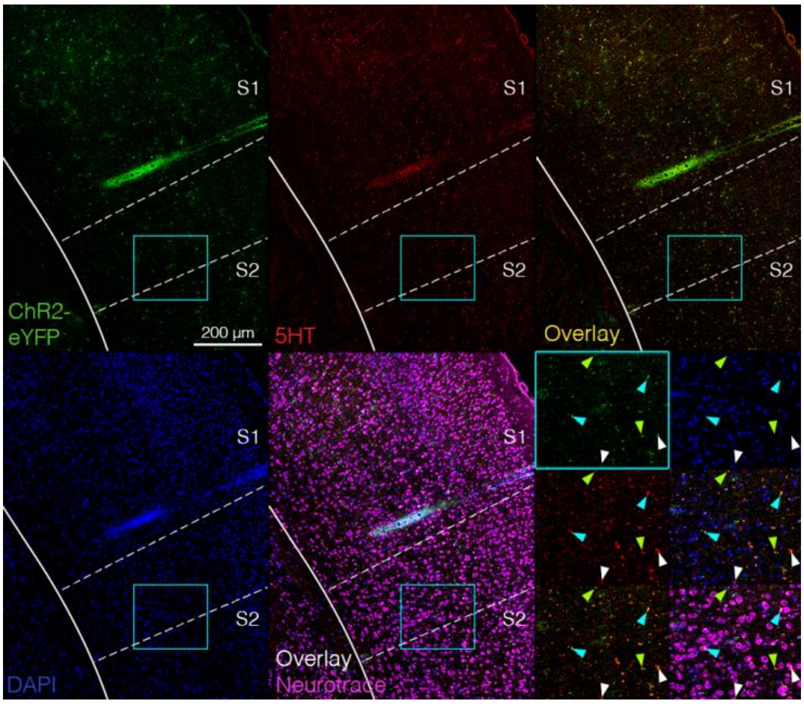

Supplementary Figure 2. Colocalisation of ChR2-eYFP and 5-HT in DRN projections to the cortex. Stitched confocal of coronal slice from an *ePet-cre*$^{+/-}$ mouse 4 weeks post-DRN-infusion of AAV harboring Ch2R-eYFP. Anti-5-HT is not expected to stain all subcellular zones of 5-HT neurons as 5-HT concentrates at axon terminals and within neuronal soma. For quantification of 5-HT and ChR2 co-staining in the DRN, see Fig. 1*C* of the main text. Arrows point to three distinct spatial locations of colocalisation. Blue: somal; White: perisomal; Green: extrasomal.

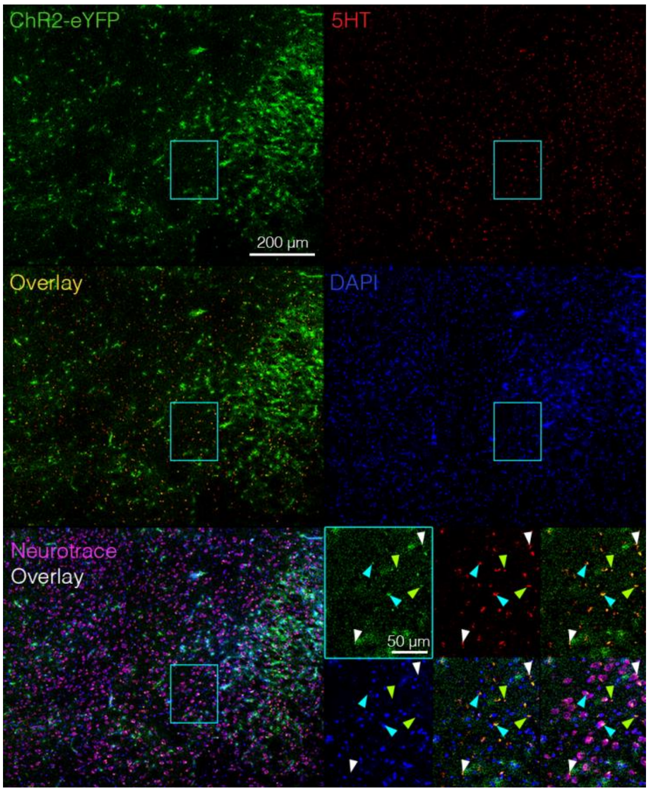

Supplementary Figure 3. Colocalisation of ChR2-eYFP and 5-HT in DRN projections to the thalamus. Stitched confocal of coronal slice from an *ePet-cre*$^{+/-}$ mouse 4 weeks post-DRN-infusion of AAV harboring Ch2R-eYFP. Anti-5-HT is not expected to stain all subcellular zones of 5-HT neurons as 5-HT concentrates at axon terminals and within neuronal soma. For quantification of 5-HT and ChR2 co-staining in the DRN, see Fig. 1*c* of the main text. Arrows point to three distinct spatial locations of colocalisation. Blue: somal; White: perisomal; Green: extrasomal.

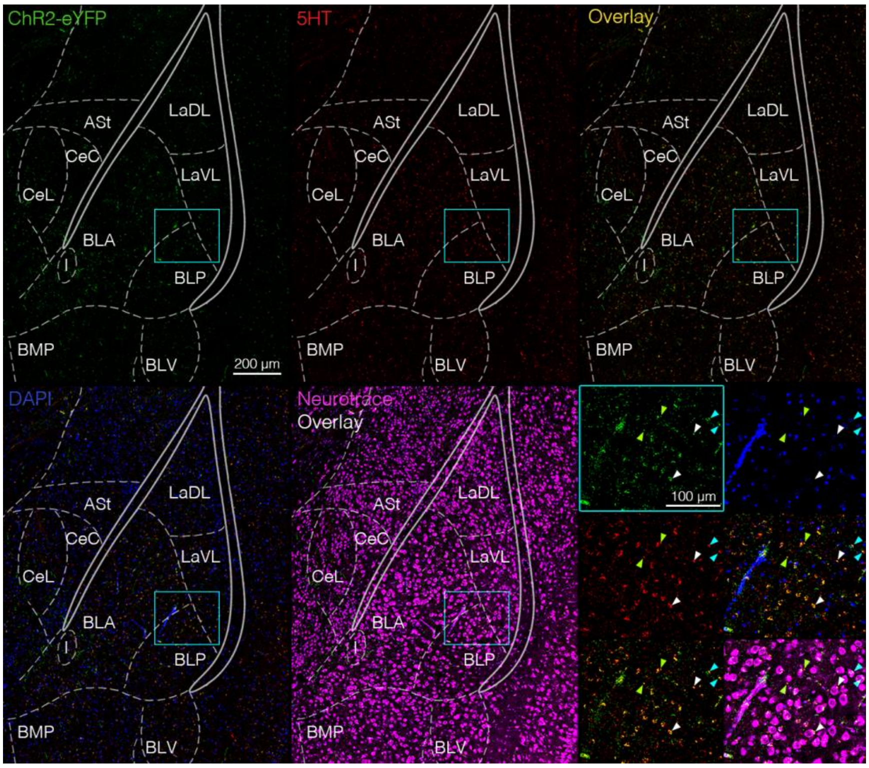

Supplementary Figure 4. Colocalization of ChR2-eYFP and 5-HT in DRN projections to the amygdala. Stitched confocal of coronal slice from an *ePet-cre*$^{+/-}$ mouse 4 weeks post-DRN-infusion of AAV harboring Ch2R-eYFP. Anti-5-HT is not expected to stain all subcellular zones of 5-HT neurons as 5-HT concentrates at axon terminals and within neuronal soma. For quantification of 5-HT and ChR2 co-staining in the DRN, see Fig. 1*c* of the main text. Arrows point to three distinct spatial locations of colocalisation. Blue: somal; White: perisomal; Green: extrasomal.

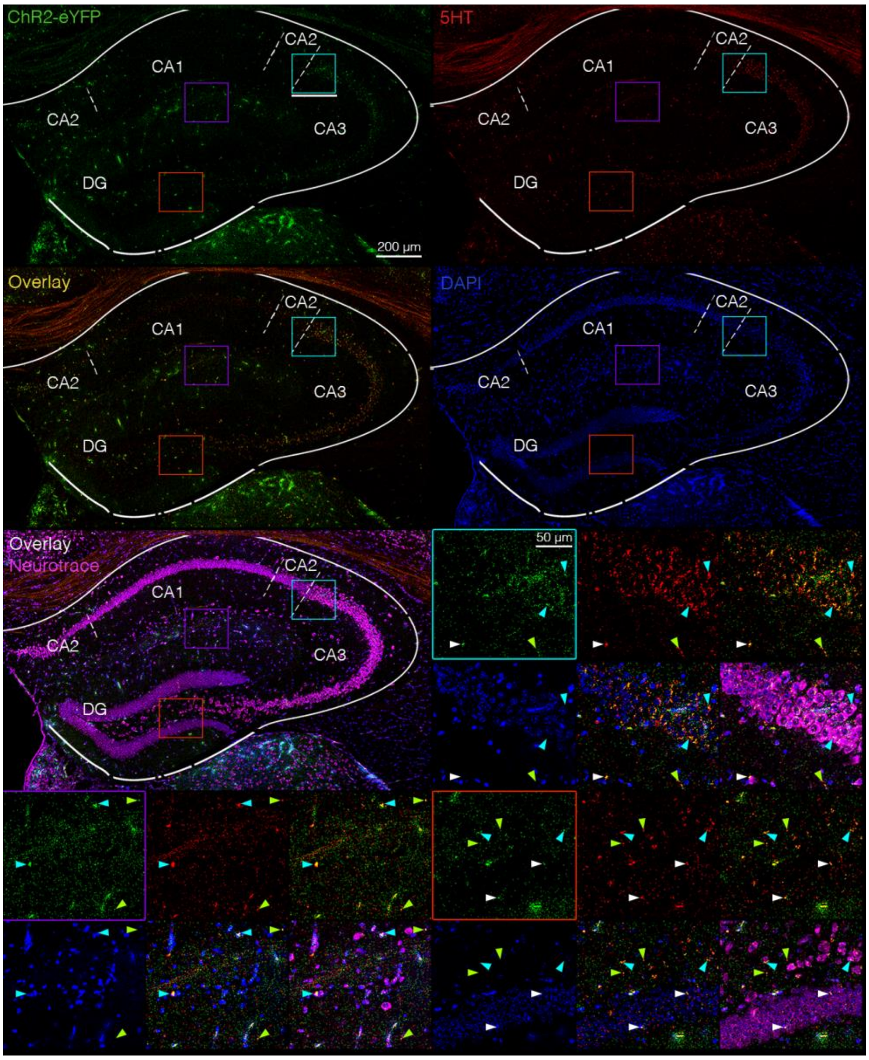

Supplementary Figure 5. Colocalisation of ChR2-eYFP and 5-HT in DRN projections to the hippocampus. Stitched confocal of coronal slice from an *ePet-cre*$^{+/-}$ mouse 4 weeks post-DRN-infusion of AAV harboring Ch2R-eYFP. Anti-5-HT is not expected to stain all subcellular zones of 5-HT neurons as 5-HT concentrates at axon terminals and within neuronal soma. For quantification of 5-HT and ChR2 co-staining in the DRN, see Fig. 1*c* of the main text. Arrows point to three distinct spatial locations of colocalisation. Blue: somal; White: perisomal; Green: extrasomal.

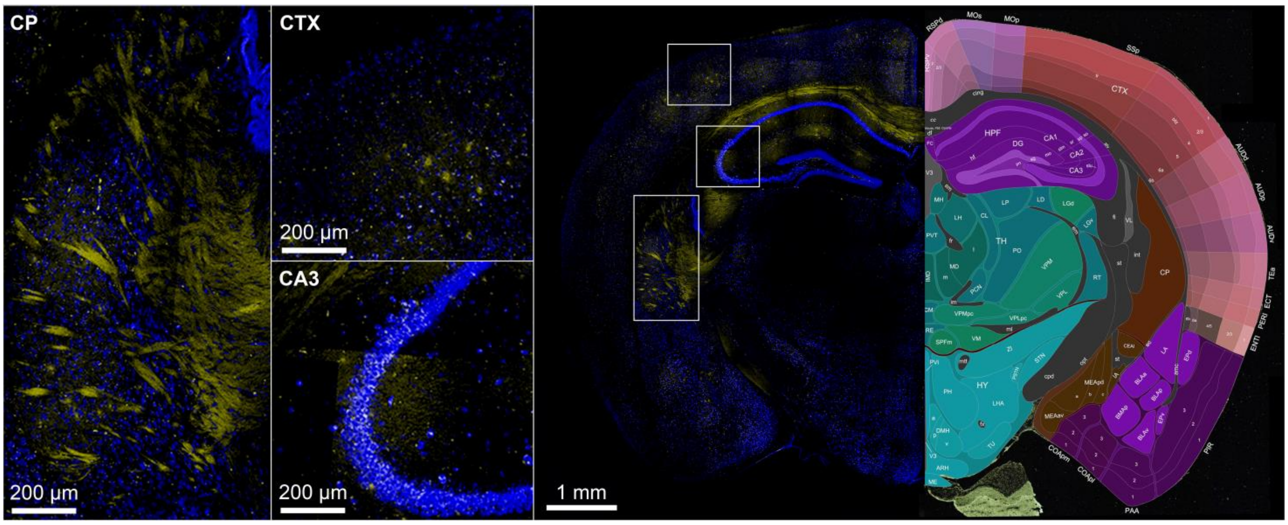

Supplementary Figure 6. Distribution of *ChR2-eYFP* (yellow) and cell nucleus (DAPI, blue) across the stitched confocal images of a coronal slice. Fluorecence is found in cortical (CTX), striatal (CP), and hippocampal (CA3) areas.

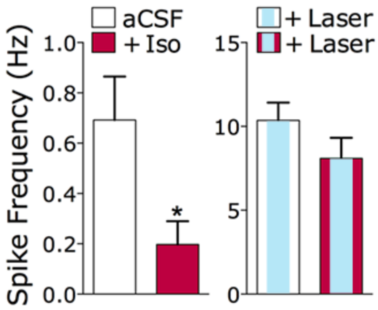

Supplementary Figure 7. Suppression of neural activity by isoflurane is overruled by ChR2-stimulation. *p<0.05 paired t-test.

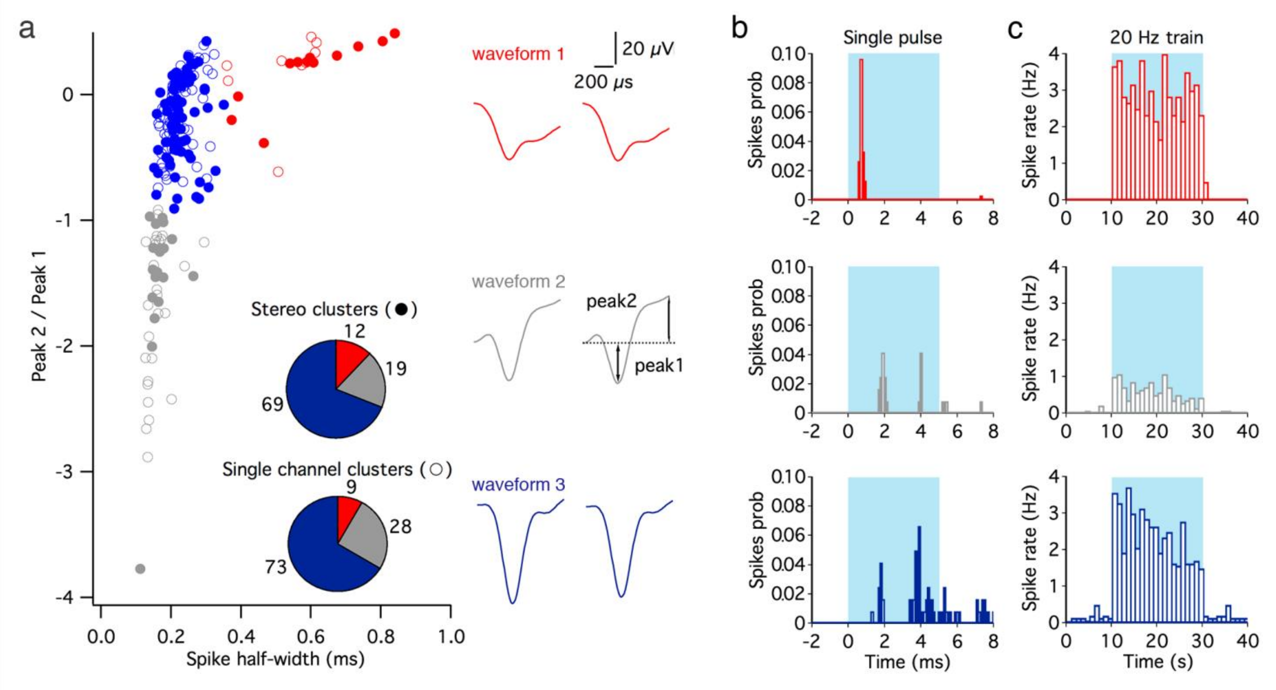

Supplementary Figure 8. (a) Segregation of three neuronal waveform clusters according to the ratio of their inward and outward peaks and their spike half-width, with the percentage of each waveform type shown in the inset pie charts. Stereo clusters refer to single units recorded from two or more electrodes. (b) Probability of each waveform cluster to spike during a 5 ms blue light pulse. (c) Spike rate of each waveform cluster before, during and after a 20 s train of blue light (20 Hz; 5 ms pulsewidth). The putative unit waveforms revealed three subtypes that could largely be distinguished based on spike half-width and rebound characteristics: type 1, characterised by a relatively broad spike waveform, type 2 neurons by a pronounced rebound, and type 3 neurons comprising the remaining majority of fast monophasic spikes. The different waveform subtypes did show some significant differences in induced spiking behavior, with type 1 neurons displaying little reverberant activity in the 10 ms following single light pulses ($p<0.05$ c.f. type 2, $P<0.001$ c.f. type 3), and type 3 neurons displayed higher spontaneous spike rates ($p<0.0001$ c.f. type 1, $P<0.01$ c.f. type 2). All subtypes exhibited delays in spike responses consistent with direct activation, and there were no significant differences in the probability of spiking in response to single low-frequency light pulses (Kruskal-Wallis=0.76, $p=0.68$) or 20 Hz pulse trains (Krustal-Wallis=1.51, $p=0.47$). Therefore, optogenetic stimulation did not appear to selectively recruit different types of DRN 5-HT neurons that could be distinguished based on their waveform.

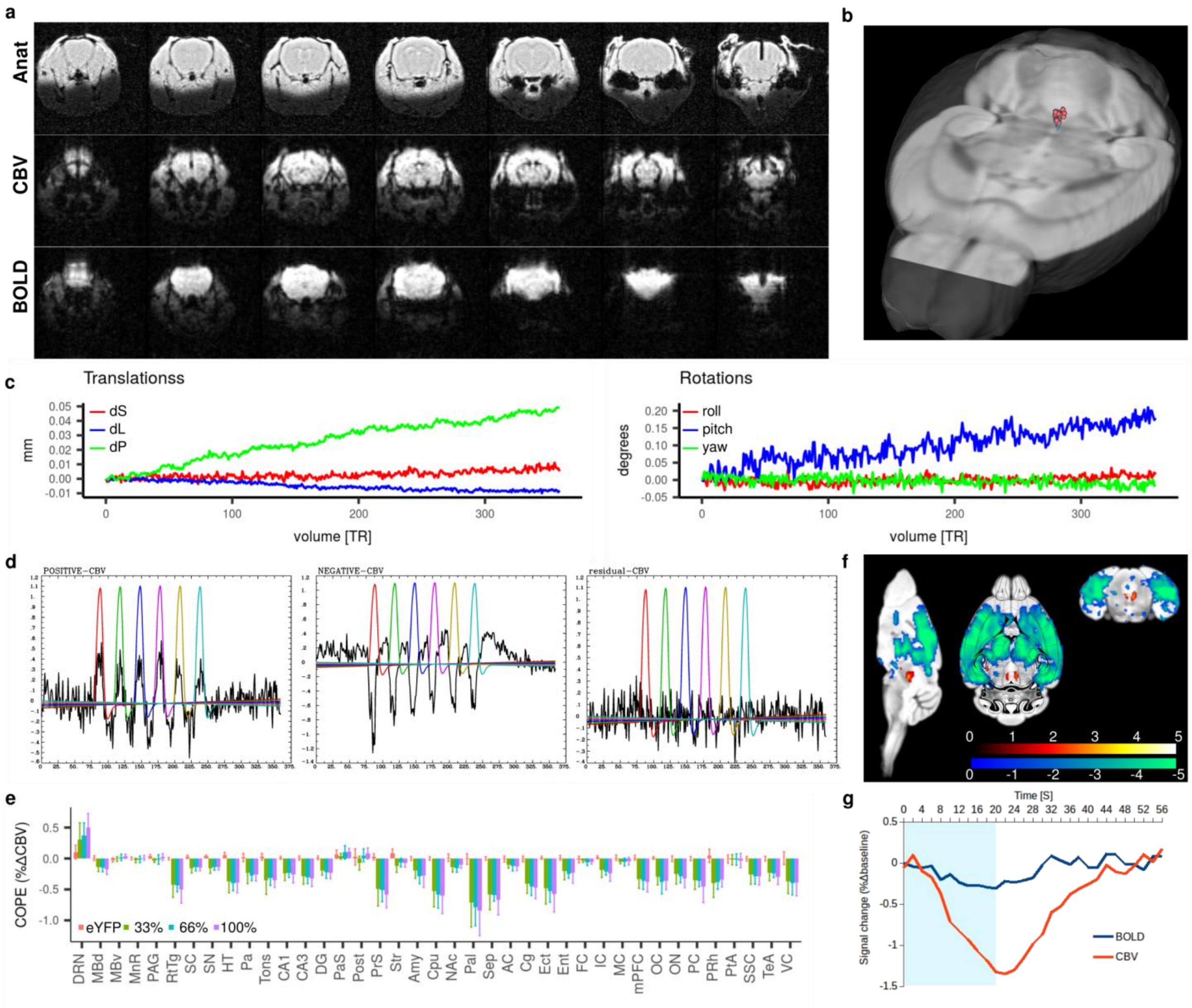

Supplementary Figure 9. (a) Representative anatomical scan denoting fiber implant in the DRN and EPI images acquired for CBV and BOLD fMRI. Images acquired for CBV presented fewer geometric distortions, specifically near ear canals, while signal from large vessels is saturated by the contrast agent. (b) Fiber implant tip (red) were poisitioned accurately with respect to the DRN (blue) in all animals included in this study. (c) Representatitive motion parameter estimates indicating rotations and translations. In some instances, a weak correlation between motion parameter estimates and stimulus paradigm was observed, attributed to image center of mass and contrast shifts due to whole-brain CBV reduction rather than animal displacement. Due to the lack of orthogonality in the design matrix, motion parameters were not included as co-variates in the GLM. (d) Convolved block design model relative to the signal extracted in voxels presenting a signficant co-activation with the model. Residual signal extracted from the same voxels indicated the variance from the block design was fully accounted. (e) COPE extracted from wild-type control animals (n=4), and animals (N=6) undergoing 3 fMRI run at

33%, 66%, and 100% laser power. No statitiscal difference (ANOVA, FDR corrected) could be not establish between the 3 power levels. Animals injected with control vector eYFP did not present detectable COPEs following 100% laser power stimulations. (f) One-sample t-test of BOLD contrast fMRI (n=8) recapitulate features observed with CBV contrast. (g) Signal extracted in the prefrontal cortex following BOLD and CBV contrast acquisition reveal a nearly 10 fold difference in response amplitude between the two modalities. Abbreviations correspond to: DRN = Dorsal raphe; MBd = Ventral midbrain; MBv = Dorsal midbrain; MnR = Median raphe; PAG = Pariacqueduct gray; RtTg = Tegmental Area; SC = Superior colliculus; SN = Substancia nigra; HT = Hypothalamus, Pa = Preoptic area; Tons = Thalamus; CA1 = Ammon's horn 1; CA3 = Ammon's horn 3, DG = Dentate gyrus; PaS = Parasubiculum; Post = post subiculum; PrS = Pre subiculum; Str = subiculum, transition area; Amy = Amygdala; Cpu = Caudate putamen (striatum); NAc = Nucleus Accumbens; Pal = Pallidum (Globus pallidus & Vantral Pallidum); Sep = Septum; AC = Auditory cortex; Cg = Cingulate; Ect = Ectorhinal cortex; Ent = Entorhinal cortex; FC = Frontal cortex; IC = Insular cortex; MC = Motor cortex, mPFC = medial prefrontal cortex; OC = Orbital cortex; ON = Olfactory nucleus; PC = Piriform cortex; PRh = Perirhinal cortex; PtA = Parietal Association cortex; SSC = Somatosensory cortex; TeA = Temporal association cortex; VC = Visual cortex

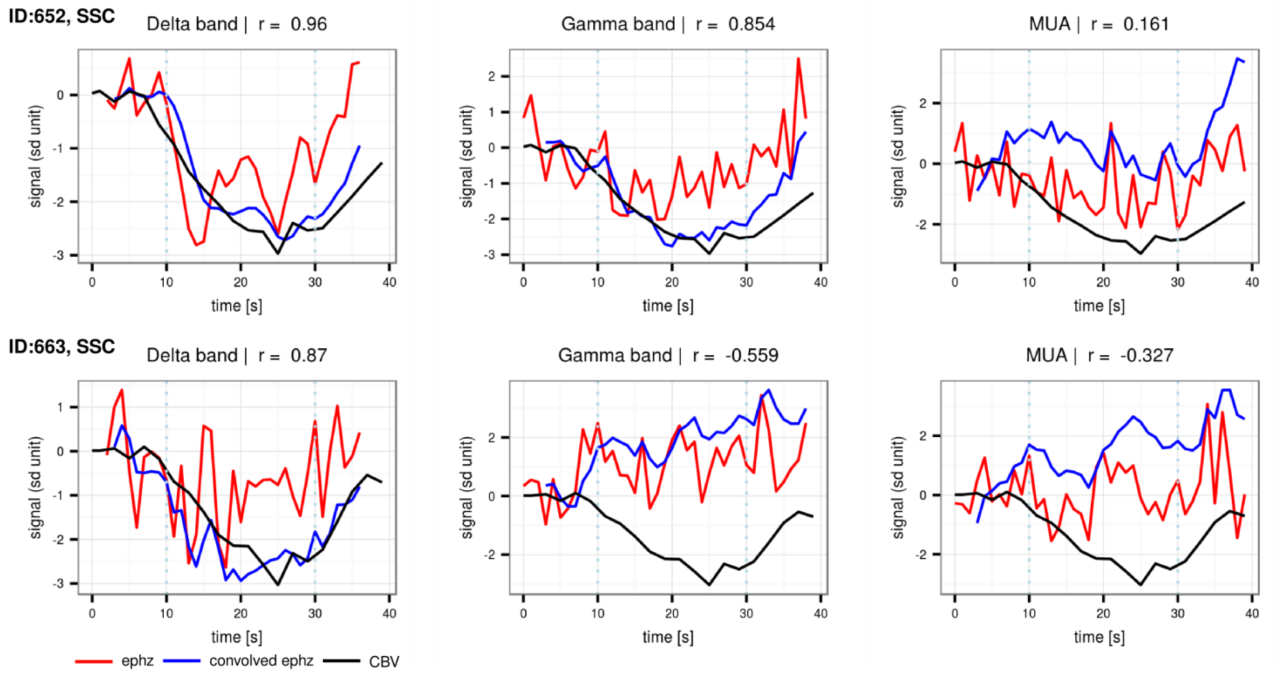

Supplementary Figure 10. Correspondance between *in*-vivo local field potential Delta and Gamma bands, and multiunit activity (MUA) with the evoked haemodynamic CBV response for two representative animals in the somatosensory cortex (SSC). The electrophysiological signal (red) was convolved with a CBV haemodynamic response function (blue). Delta band presented strongest correlations with the CBV response (r = 0.75 ± 0.16, n=6 recordings).

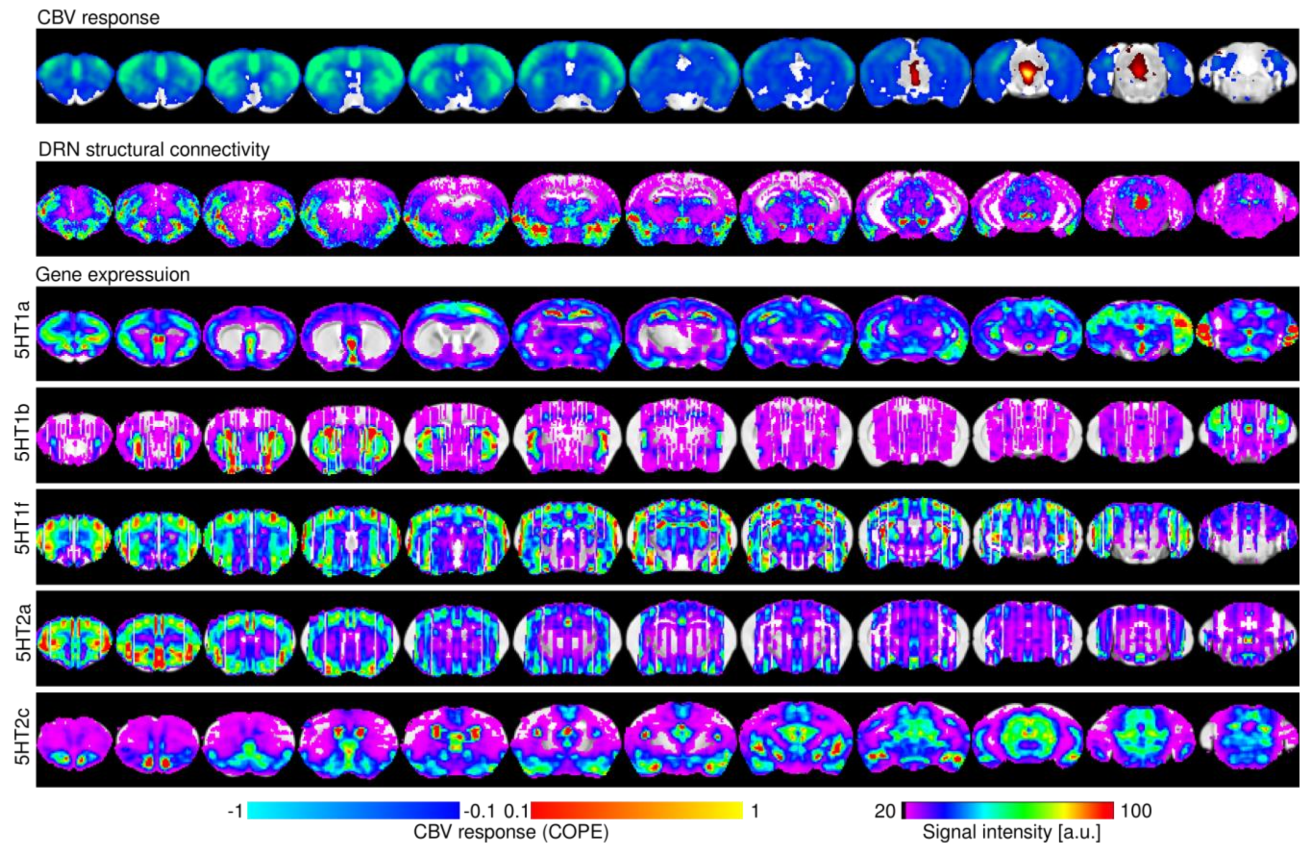

Supplementary Figure 11. Mean COPEs derived from the voxel-wise analysis. DRN projections and 5HT receptor gene expressions obtained from Allen Brain Institute database, experiment IDs: 114155190 (DRN projections), 79556616 (5HT1a), 583 (5HT1b), 69859867 (5HT1f), 81671344 (5HT2a), 73636098 (5HT2c).

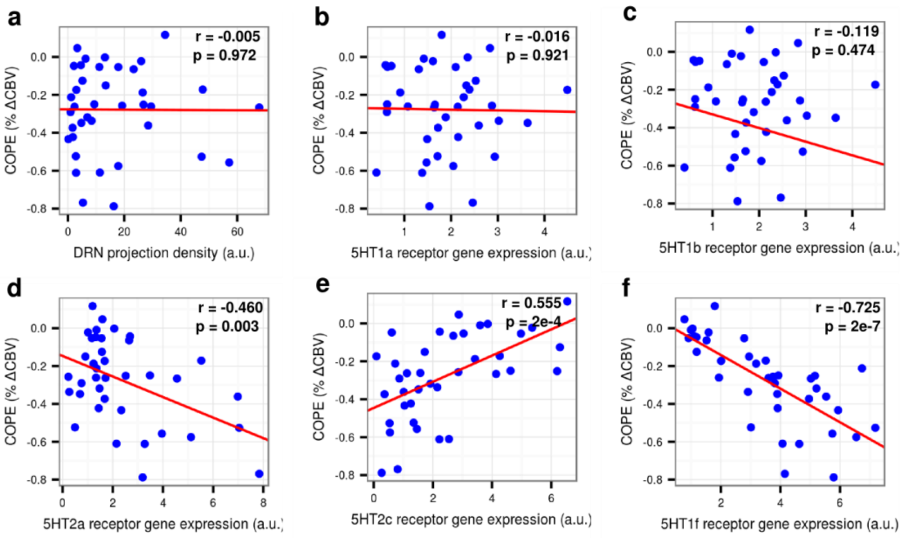

Supplementary Figure 12. Correlations between group averaged COPEs, DRN projection field derived, and 5HT receptor gene expressions.

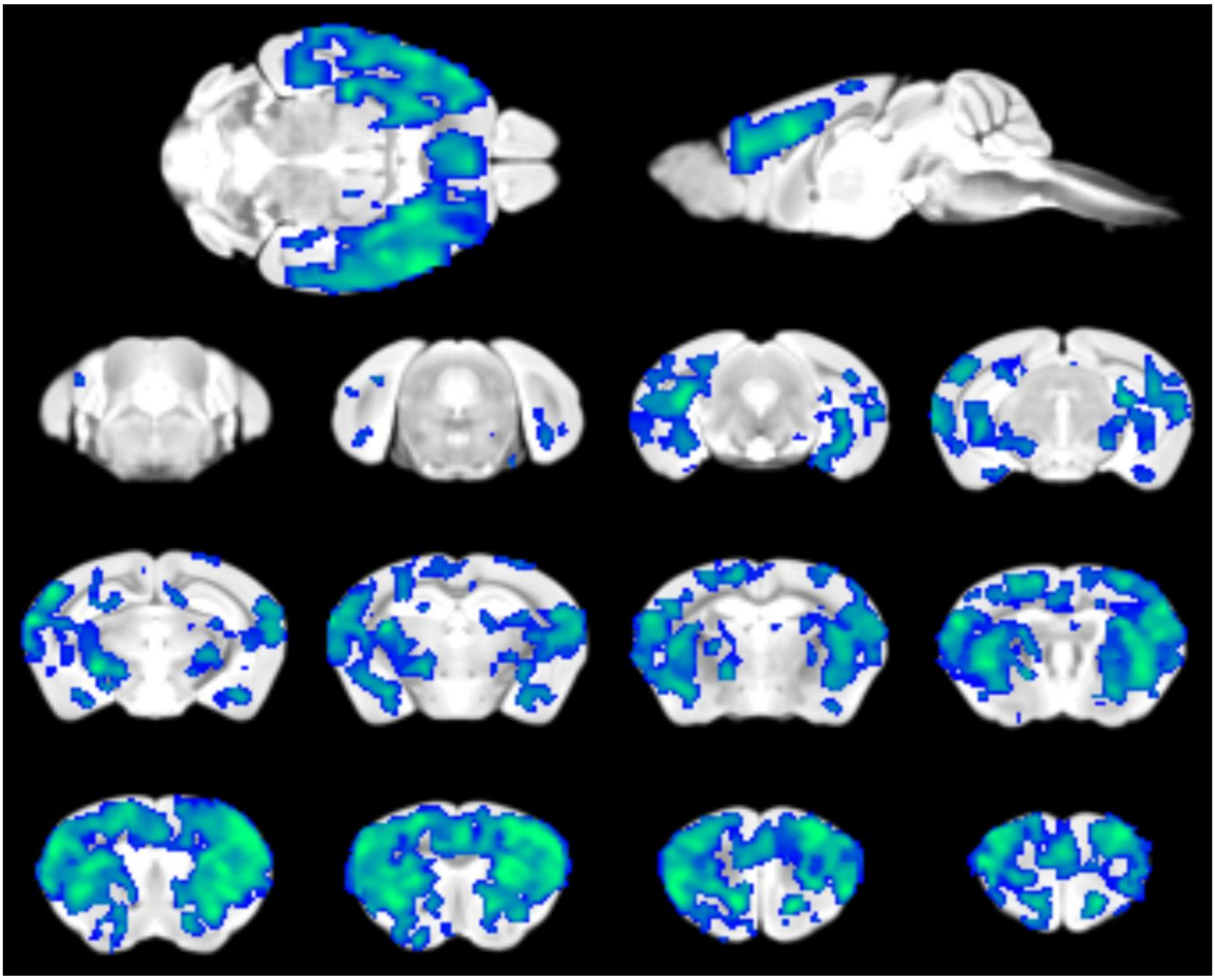

Supplementary Figure 13. Second level voxel-wise GLM for comparison restraint (n = 7) > control (n = 4), thresholded at p≤0.05 (cluster corrected).

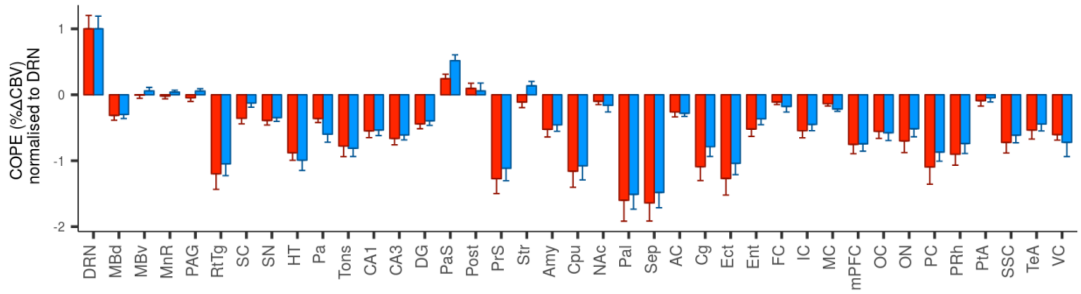

Supplementary Figure 14. ROI COPEs for restraint vs control normalised to the DRN.

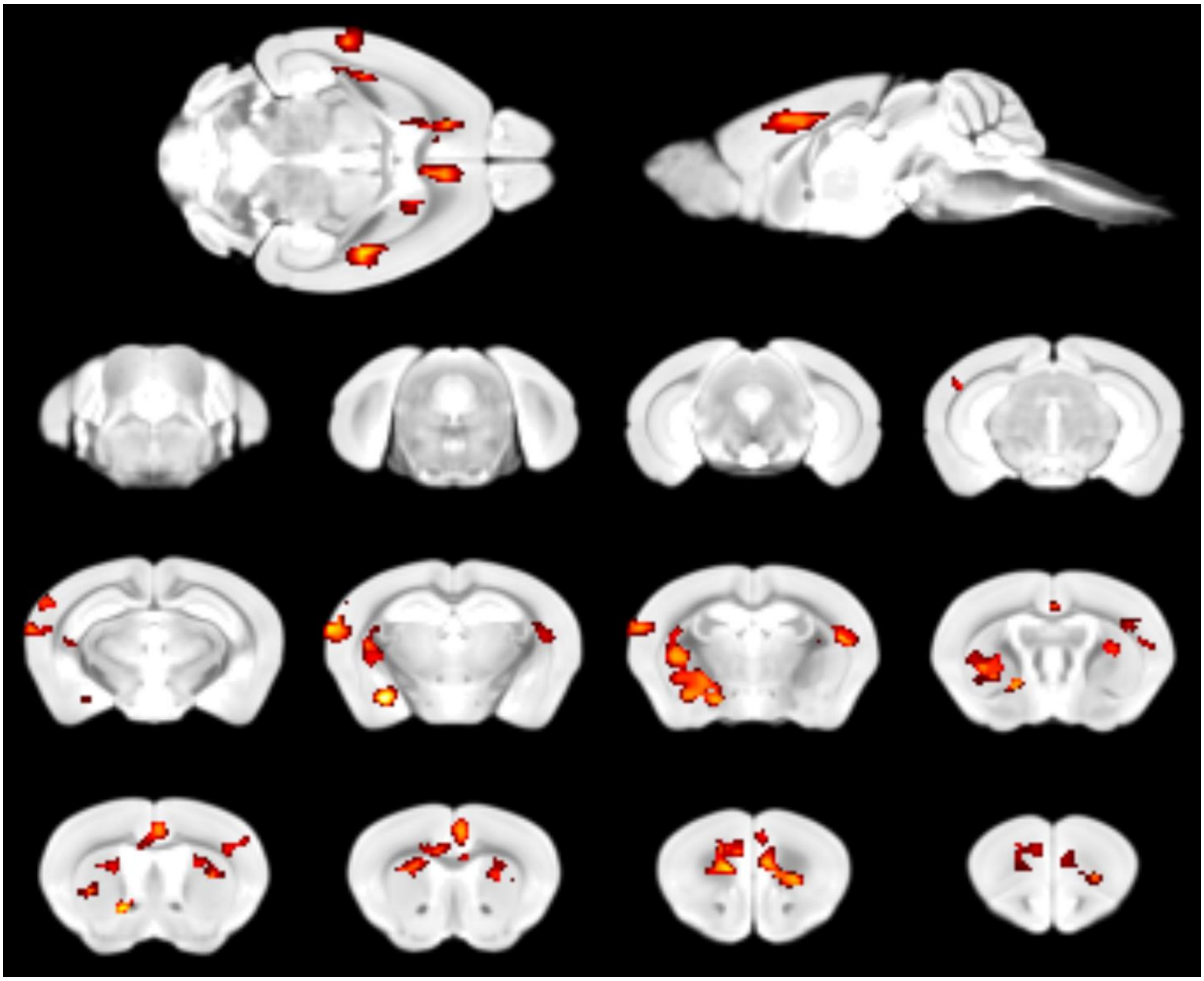

Supplementary Figure 15. Second level analysis for comparison fluoxetine (n=9) > control (n=4), thresholded at $p \leq 0.05$ (cluster corrected).

Supplementary Table 1 | Antibodies and other markers

Antigen / Target	Regent	Source	Conditions	RRID
5-HT	rabbit anti-5-HT	Abcam	1:2000 in 1% horse serum: O/N @ R°T	AB_297124
Neurons	NeuroTrace Red	Molecular Probes	1:20 in 1% horse serum; 20 min @ R°T	n/a
DNA (Nuclear stain)	DAPI	Sigma-Aldrich	0.02 µg/ml in PBS; 2 min @R°T	n/a
DNA (Nuclear stain)	TO-PRO-3	Molecular Probes	0.07 µg/ml in PBS; 5 min @R°T	n/a
rabbit anti-5-HT	CY5 donkey anti-rabbit IgG	Jackson ImmunoResearch Labs	1:400 in 1% horse serum; 2 h @ R°T	AB_2340607
rabbit anti-5-HT	CY3 donkey anti-rabbit IgG	Jackson ImmunoResearch Labs	1:400 in 1% horse serum; 2 h @ R°T	AB_2307443
GFP	mouse anti-GFP	BioSystems	1:200 in 1% horse serum; 2 h @ R°T	AB_11158995

Supplementary Table 2 | List of stereotaxic coordinates

Target Region	Purpose	Bregma*	Lambda*	Midline**	Angle***	Dorsal*†
Dorsal raphe	Virus infusion	n/a	-0.6	+1.0	20°	-3.6 (rSkull)
Dorsal raphe	Optic fiber implant	n/a	-0.6	0.0	0°	-3.3 (rSkull)
Dorsal raphe	MUA & LFP probe placement	n/a	-0.3 to -0.9	+1.5 to +2.5	15° to 30°	-3.0 to -4.5
M1	MUA & LFP probe placement	-1.0 to +1.0	n/a	+1.3 to +2.0	0°	-1.2
S1	MUA & LFP probe placement	-2.0 to +1.0	n/a	+1.5 to +2.7	0°	-1.2
HPF	MUA & LFP probe placement	-2.0 to -2.5	n/a	+1.5 to +2.0	0°	-2.0
Insular cortex	MUA & LFP probe placement	+1.0	n/a	+3.0	0°	-1.2
Cingulate/M2	MUA & LFP probe placement	-1.0 to +1.0	n/a	+1.0	10°	-2.0
FrA	MUA & LFP probe placement	+3.0	n/a	+1.5	0°	-1.2

*Coordinates provided in mm
**Coordinates provided right of midline
***Angle from normal
†Relative to dura, unless otherwise stated
rSkull = relative to surface of skull
M1/2=motor cortex area 1/2; S1=Sensory cortex area 1; HPF=Hippocampal formation; FrA=Frontal association cortex
